# Dissecting the inhibitory activity of *Burkholderia orbicola* against Gram-positive and - negative multidrug-resistant bacteria

**DOI:** 10.1371/journal.pone.0326906

**Published:** 2025-06-30

**Authors:** Leslie-Mariana Morales-Ruíz, Anuar Salazar-Gómez, Álvaro-Omar Hernández-Rangel, Jeniffer-Chris Kerber-Díaz, María-Elena Vargas-Díaz, J. Antonio-Ibarra, Fernando-Uriel Rojas-Rojas, Paulina Estrada-de los Santos

**Affiliations:** 1 Departamento de Microbiología, Escuela Nacional de Ciencias Biológicas, Instituto Politécnico Nacional, Ciudad de México, México; 2 Escuela Nacional de Estudios Superiores Unidad León, Universidad Nacional Autónoma de México, León, Guanajuato, México; 3 Departamento de Química Orgánica, Escuela Nacional de Ciencias Biológicas, Instituto Politécnico Nacional, Ciudad de México, México; 4 Laboratorio de Ciencias AgroGenómicas, Escuela Nacional de Estudios Superiores Unidad León, Universidad Nacional Autónoma de México, León, Guanajuato, México; 5 Laboratorio Nacional PlanTECC, Escuela Nacional de Estudios Superiores Unidad León, Universidad Nacional Autónoma de México, León, Guanajuato, México; University of Buea, CAMEROON

## Abstract

The genus *Burkholderia* is currently recognized for producing several antimicrobial compounds with potential applications in developing novel treatments for infectious diseases, including those caused by multidrug-resistant (MDR) bacteria. This study aimed to investigate the ability of *Burkholderia orbicola* TAtl-371^T^ and CACua-24 to inhibit the growth of MDR human pathogens and to analyze the chemical composition of active extracts from cultures of these strains to identify putative compounds associated with their activity. The double-layer agar technique was used to evaluate the antimicrobial activity of *B. orbicola* strains. Sequential solvent extraction with hexane, dichloromethane, ethyl acetate, and methanol was conducted on *B. orbicola* cultures, and the active extract was analyzed by bioautography and fractionated using preparative thin-layer chromatography. Putative antimicrobials in the active fraction were identified through ^1^H, ^13^C NMR, and mass spectrometry. *B. orbicola* strains inhibited several MDR strains of *Acinetobacter baumannii*, *Klebsiella pneumoniae*, *Pseudomonas aeruginosa*, *Escherichia coli*, and *Staphylococcus aureus* on double-layer agar probes, displaying inhibition halos ranging from 5 to 41 mm. The hexane extract showed the best inhibitory activity against MDR strains, except for *P. aeruginosa* strains. Analysis through thin-layer chromatography and bioautography revealed a tailing spot with antimicrobial activity. The spectroscopic analysis of this tailing spot revealed the presence of the siderophore fragin. This fragin-containing fraction inhibited the MDR *A. baumannii* (1024 µg/mL), *K. pneumoniae* 903137 (128 µg/mL), *E. coli* (256 µg/mL), and *S. aureus* (128 µg/mL), but no effect was observed against *P. aeruginosa*. This fraction also inhibited yeasts of the species *Candida albicans* and *Nakaseomyces glabratus*, suggesting an antimicrobial spectrum that extends beyond MDR bacteria. The genomic sequence analysis of strains TAtl-371^T^ and CACua-24 revealed a cluster of 7 genes, resulting in the same organization and over 99% similarity to the fragin genes reported for *Burkholderia cenocepacia* H111. This study highlights the potential of *B. orbicola* to produce fragin and its potential activity against MDR bacteria that affect human health worldwide.

## Introduction

The development of antibiotic resistance by pathogenic bacteria is one of the major global healthcare threats. Infections caused by several multidrug-resistant (MDR) Gram-positive and Gram-negative bacteria, which lead to increased mortality and morbidity, pose a challenge to treatment with traditional drugs [1]. Consequently, the World Health Organization (WHO) redacted and published a list of priority MDR pathogens, updated annually [2]. This list aims to encourage researchers worldwide to increase the studies looking for new drugs to address the health issues caused by MDR bacteria, such as those in the ESKAPE group (*Acinetobacter baumannii*, *Pseudomonas aeruginosa*, *Escherichia coli*, and *Staphylococcus aureus*, among others). In 2021, the WHO reported that none of the 43 antibiotics in clinical development would effectively address the drug-resistance problem [3], emphasizing that these antibiotics are just variations of molecules discovered decades ago. Thus, within the strategies to combat antibiotic resistance, the investigation of new natural products produced by microorganisms remains a promising strategy for finding bioactive compounds that can address the problems caused by MDR [4]. *Burkholderia* is a highly recognized genus that produces specialized metabolites (SM) with antibacterial, antifungal, antitumor, antiviral, or immunosuppressive activity [5]. The antimicrobial compounds of *Burkholderia* are produced by different biosynthetic pathways, which could include non-ribosomal peptide synthetases (NRPS), polyketide synthetases (PKS), hybrids of the two previous (NRPS-PKS), or through other specialized pathways to produce bacteriocins, non-ribosomal peptides, and polyketides [6]. The analysis of SM from described *Burkholderia* strains shows that 50% are non-ribosomally produced compounds, either NRPS (13%), hybrid NRPS-PKS (23.7%), and trans-AT(acyl transferases)-PKS (13.1%) [6].

Our previous work demonstrated the antimicrobial activity of *Burkholderia orbicola* TAtl-371^T^ against bacteria, fungi, and yeast, including both environmental and clinical strains [7,8]. The genome analysis at that time revealed potential genes involved in the metabolic pathways for synthesizing siderophores, bacteriocins, NRP, and chitinases that might have antimicrobial activity [7,9]. We also observed the role of siderophores production in the antagonism of TAtl-371^T^ against *Candida glabrata* (now *Nakaseomyces glabratus*) and *Paraburkholderia phenazinium*, as well as the bactericidal effect of a lectin-like bacteriocin 88 (LlpA88) on some species within the *Burkholderia cepacia* complex (Bcc) [7]. The reference strain *B. orbicola* CACua-24 was also studied in our group to produce antifungal metabolites that inhibited *Rhizoctonia solani*, *Candida albicans*, *Pythium ultimum,* and Gram-negative bacteria [8,10]. However, most of the antimicrobial compounds responsible for the broad spectrum of antimicrobial activity of both *B. orbicola* strains remain to be elucidated. Currently, we are attempting to identify these molecules produced by *B. orbicola* TAtl-371^T^ and CACua-24, especially the ones that could serve as suitable molecules for treating MDR bacterial human pathogens. Here, we report the identification of the siderophore fragin in hexane extracts from agar cultures of *B. orbicola* with activity against the growth of MDR strains of *S. aureus*, *A. baumannii*, *E. coli,* and *Klebsiella pneumonia*e, some of the priority pathogens for which new and effective antibiotics should be developed in the coming years.

## Materials and methods

### Microbial strains

The antimicrobials-producing strains were *B. orbicola* TAtl-371^T^ and CACua-24 [8]. The indicator bacteria were the MDR strains of *A. baumannii* (strains 256, 324, 341, 344, 345), *K. pneumoniae* (strains 97833, 9851043, 81739, 945626, 906667, 903137), *P. aeruginosa* (strains 1P, 2P, 4P, 11P, 12P, 16P, 17P, 22P, 26P, 30P), *E. coli* (strains 1, 2, 3, 4), and *S. aureus* (strains 1, 2, 3, 4) provided by Dr. Graciela Castro-Escarpulli (ENCB-IPN). *Tatumella terrea* SHS 2008^T^ was used as a positive control due to its high sensitivity to *B. orbicola* antagonism [7]. This bacterium allowed us to ensure the production of *B. orbicola* antimicrobials on PDA and the presence of these compounds in extracts and fractions. The MDR strains were characterized for antibiotic susceptibility using the VITEK system and AST N271 card for Gram-negative bacteria and AST GPT for Gram-positive bacteria (S1 Table). *C. albicans* (strains ATCC 10231 and 30) and *N. glabratus* (strains CBS 138 and 43) were provided previously by Dr. Lourdes Villa Tanaca (ENCB-IPN) and used only to analyze the antimicrobial spectrum beyond the inhibition of bacteria. Strains were routinely grown in LB, potato dextrose agar, or broth (PDA or PDB, respectively) at 30°C. All strains were stored in 35% glycerol at −70°C.

### Antimicrobial activity assays

The antimicrobial activity of *B. orbicola* strains was detected using the double-layer agar technique [7]. Briefly, bacterial strains TAtl-371^T^ and CACua-24 were spotted (2 μL from an overnight culture, approximately 1 x 10^9^ CFU) in the center of a PDA plate and incubated at 30°C for 72 h. Next, the plates were exposed to chloroform vapors and overlaid with a soft agar medium seeded with the indicator strain (positive control or the MDR strains).

For extracts and fractions, 100 μg (for *T. terrea* SHS 2008^T^) and 200 μg (for each MDR) were dissolved in 5 μL of isopropyl alcohol and added to sterile paper filters (5 mm) in a disc diffusion test, allowed to evaporate the solvent under sterile conditions and placed on soft PDA plates seeded with the indicator strain. The plates containing MDR strains were incubated for 24 h at 37°C, and *T. terrea* SHS 2008^T^ (positive control) was incubated for 48 h at 30°C, according to the optimal growth conditions of each microorganism. The inhibition zones were measured at the end of the incubation time. The experiments were carried out in duplicate.

### Preparation of the extracts and antimicrobial activity

*B. orbicola* TAtl-371^T^ and CACua-24 were spotted thrice (2 μL each) on PDA plates (15 plates) and incubated at 30°C for 72 h. After the incubation, the bacterial colonies were removed with filter paper. The agar was sliced with a sterile scalpel into small square pieces, detached, and placed in a 1 L flask. It was then extracted sequentially with hexane (Hex), dichloromethane (DCM), ethyl acetate (EtOAc), and methanol (MeOH). The extracts were named as follows: for TAtl-371^T^ with Hex = EHexT, with DCM = EDCMT, with EtOAc = EEtOAcT and with MeOH = EMeOHT; and for CACua-24 with Hex = EHexC, with DCM = EDCMC, with EtOAc = EEtOAcC and with MeOH = EMeOHC. Each extraction step was conducted three times using 300 mL of each solvent in individual batches, which were combined and evaporated under reduced pressure with a rotary evaporator (Scilogex). To test antimicrobial activity on *T. terrea* SHS 2008^T^ (positive control) and MDR strains, 100 μg or 200 μg of each extract were suspended in 5 μL of isopropyl alcohol and used as described in the previous section. As a negative control, extracts from uninoculated PDA were obtained following the same procedure.

### Thin-layer chromatography bioautography for screening of active fractions

Analytical thin-layer chromatography (TLC) of hexane extracts from TAtl-371^T^ (EHexT) and CACua-24 (EHexC) was performed using precoated TLC plates with silica gel 60-F_254_ (Merck), Hex:EtOAc (1:1 v/v) and Hex:DCM:MeOH (3:10:2 v/v) as mobile phases and visualized by UV detection at 254, 302, and 365 nm. Bioactive spots were identified with a bioautography test [11]. For this assay, the TLC plates were placed in a Petri dish and covered with 6 mL of soft PDA (0.5% agar) inoculated with the positive control strain (*T. terrea* SHS 2008^T^). The plates were incubated at 30°C for 48 h. Following the incubation period, the plates were overlaid with 6 mL of soft PDA mixed with 200 µL of 5 mg/mL 3-(4,5-dimethylthiazo-2-lyl)-2,5-diphenyltetrazolium bromide (MTT) and incubated at room temperature in the dark for 30 min. The zone of the TLC exhibiting no purple color was considered the active fraction, and the Rf value was measured and calculated. To identify putative functional groups in the molecules present in the active fraction, the TLC plates were stained with ninhydrin (2%) and Salkowski reagent to screen for the presence of amino group-containing compounds [12] and indole compounds [13], respectively.

### Chemical composition analysis of active fraction

Preparative glass TLC silica gel 60 plates F_254_ (20 cm x 20 cm) (Merck) were used to obtain the active fraction of EHexT and EHexC. For this, 15 mg of each dried extract was resuspended in DCM and spotted on a horizontal line on the preparative TLC plates. The samples on the preparative TLC plates were separated with a solvent system of Hex:EtOAc (1:1 v/v). After visualization under UV light (302 nm), the active fraction (a tailing spot) was divided into four subfractions, scraped from the TLC, and collected in glass vials. The subfractions were extracted from the silica gel using DCM and EtOAc (1:1), and then filtered through a 0.45 μm sterile filter. Each subfraction was evaporated to dryness under reduced pressure and then tested for activity as described above.

The biologically active subfractions of the tailing spot of EHexT were mixed, labeled as fraction D, and analyzed by nuclear magnetic resonance (NMR) spectroscopy and mass spectrometry analysis to identify mainly compounds. The compound identification was supported by directly comparing their spectral data and ion peaks with those previously reported [15,16]. NMR spectra were obtained on the NMR spectrometer Bruker Ascend 600, Bruker, Germany (600 MHz for ^1^H and 150 MHz for ^13^C) at 10°C using 6.35 mg/mL of the active fraction D of EHexT and CDCl_3_ as solvent. Chemical shifts (δ) are reported in parts per million (ppm) relative to internal tetramethylsilane (Me_4_Si, *δ* 0.0) for ^1^H NMR and CDCl_3_ (*δ* 77.0) for ^13^C NMR. Coupling constants (*J*) are reported in Hertz (Hz). Multiplicities are indicated by s (singlet), d (doublet), ddd (doublet of doublet of doublets), t (triplet), td (triplet of doublets), and m (multiplet). Mass spectrometry analysis was performed in a Bruker MicrOTOF-QII system by an electrospray ioniza-tion (ESI) interface (Bruker Daltonics, Billerica, MA, USA) operating in the positive ion mode. After the observed chemical analysis results, a qualitative test was carried out on EHexT to confirm the presence of the diazeniumdiolate functional group [14]. For this, 20 mg of EHExT was added to 500 µL of phenol and heated for 2 min at 45°C. Then, 4–6 drops of H_2_SO_4_ were added to the mixture to reveal the presence of the functional group as a development of red color [14].

### Inhibitory spectrum of active fraction D

The active fraction D of EHexT was tested against MDR bacteria using the double-layer antagonism assay described above. To identify a spectrum of activity beyond MDR bacteria, we also tested the active fraction D against yeasts. In brief, 200 μg of fraction D was dissolved in 5 μL of isopropyl alcohol and placed on a filter paper disc. Once the solvent was evaporated, the disc was placed in the center of a PDA plate. Then, a layer of soft agar inoculated with each MDR strain or yeast was overlaid, and the plates were incubated at 37°C for 24 h.

### Minimum inhibitory concentration of active fraction D

The minimum inhibitory concentration (MIC) of the active fraction D of EHexT against MDR strains and yeast was determined according to the Clinical & Laboratory Standards Institute [17] using 96-well plates. The indicator strains were resuspended in a Mueller-Hinton (MH) liquid medium to achieve a 0.5 McFarland turbidity [17]. Then, 50 μL were deposited in different wells containing fraction D dissolved in 3% DMSO at final concentrations of 1024, 512, 256, and 128 μg/mL. The indicator strains growing on MH were used as a mock. As a negative control, a mixture of MH and fraction D, without the inoculation of any microorganism, was used. The plates were incubated at 37ºC for 24 h (150 rpm). The microbial growth was measured at 600 nm using a multiscan (ThermoScientifc). All experiments were performed with three replicates on different days, using the fraction D obtained from the same culture.

### Active fraction D toxicity test in a *Galleria mellonella* model

The assay was performed using *Galleria mellonella* larvae at the last instar stage, approximately 3 cm in length [18]. A group of 10 larvae was injected with 10 µL of active fraction D diluted in 30% DMSO to achieve concentrations of 50, 100, 200, and 300 μg/mL. The negative control consisted of 10 larvae injected with 10 µL of 30% DMSO [19]. For the positive control, serial dilutions were performed from a 24 h culture of *B. orbicola* TAtl-371^T^ in LB medium, up to 1 x 10^−5^, equivalent to approximately 8.2 x 10^7^ CFU for the inoculum (10 µL) of the corresponding group of larvae [10]. The groups of larvae were kept in Petri dishes with a piece of filter paper (8 cm diameter) at 25 ºC for 72 h. After the incubation time, the mortality rate of the larvae was assessed. The experiment was performed with three replicates.

### Fragin biosynthetic genes

The genes for the biosynthesis of fragin were analyzed in *B. orbicola* strains using the cluster described by Jenul et al. [14], corresponding to *hamABCDEFG* genes. The genes correspond to the following proteins: HamA, Haem-oxygenase-like, multi-helical; HamB, RmlC-like cupin domain superfamily; HamC, p-aminobenzoate N-oxygenase AurF; HamD consists of an adenylation domain, thiolation domain, and reductase domain; HamE, polyketide cyclase/dehydrase; HamF, condensation domain; HamG, aminotransferase class-III.

## Results

### Inhibition of MDR strains

**Fig 1:**
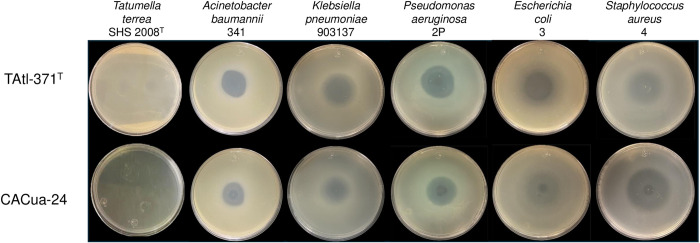
Antimicrobial activity of *Burkholderia orbicola* TAtl-371^T^ and CACua-24 against multidrug-resistant bacterial strains using the double-layer agar technique. *Tatumella terrea* SHS 2008^T^ was used as a positive control.

*Burkholderia orbicola* TAtl-371^T^ and CACua-24 were tested against MDR strains using the double-layer agar technique. The metabolites produced by both strains impeded the growth of the MDR bacteria with clear inhibition halos around the inoculated spot (Fig 1). Indicator strains inhibited by *B. orbicola* were all *A. baumannii, K. pneumoniae, P. aeruginosa,* and *S. aureus* strains (S1 Fig). The most strongly inhibited bacteria were *P. aeruginosa* and *A. baumannii,* the least sensitive was *K. pneumoniae,* and the biggest inhibition halos were observed in *B. orbicola* CACua-24 (S2 Table). Strain 4 from *E. coli* was not inhibited by *B. orbicola* TAtl-371^T^, but the rest of the strains were inhibited by both TAtl-371^T^ and CACua-24 (S1 Fig). The inhibition of *T. terrea* SHS 2008^T^, a susceptible strain to TAtl-371^T^ [7], was almost complete with *B. orbicola* TAtl-371^T^ (70 mm) and entirely with *B. orbicola* CACua-24 since no growth was observed in the agar plates (Fig 1).

### Antimicrobial activity of extracts

**Fig 2:**
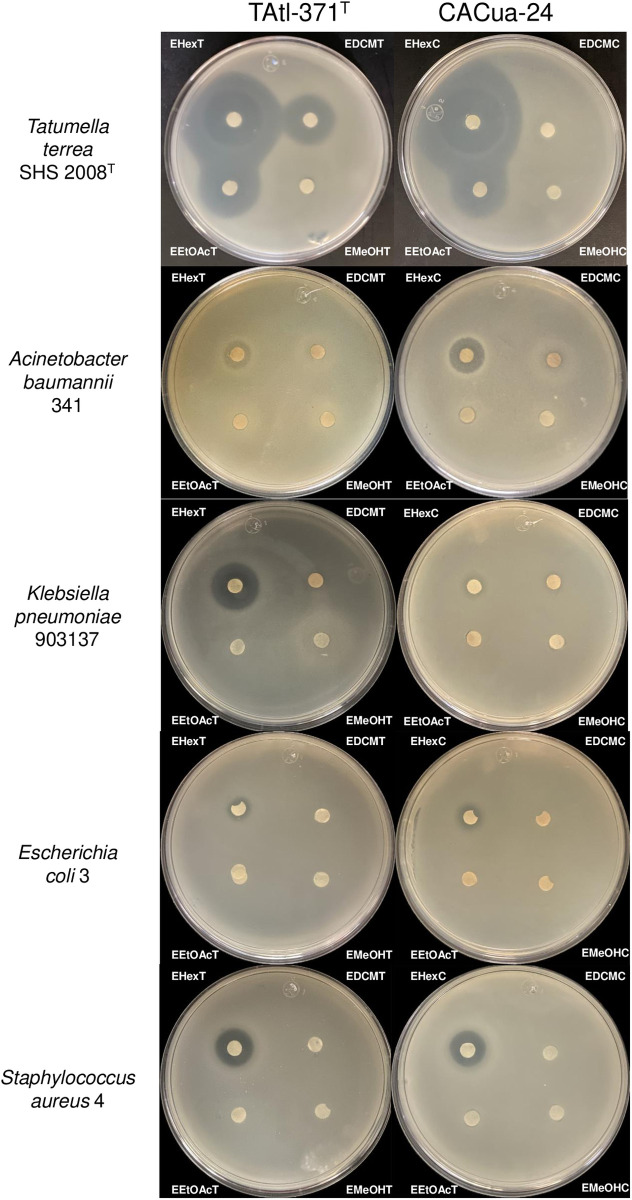
Antimicrobial activity of hexane (Hex), dichloromethane (DCM), ethyl acetate (EtOAc), and methanol (MeOH) extracts obtained from *Burkholderia orbicola* TAtl-371^T^ and CACua-24 cultures against *Tatumella terrea* SHS 2008^T^ (100μg) and multidrug-resistant bacteria (200 μg) using the double-layer agar technique. The extracts were named as follows: for TAtl-371^T^ with Hex = EHexT, with DCM = EDCMT, with EtOAc = EEtOAcT and with MeOH = EMeOHT; and for CACua-24 with Hex = EHexC, with DCM = EDCMC, with EtOAc = EEtOAcC and with MeOH = EMeOHC.

Antimicrobial compounds produced by strains TAtl-371^T^ and CACua-24 were extracted from PDA plates using different solvents from 72-hour-old cultures. Extracts of uninoculated PDA plates were used as a control. After the extraction procedure from TAtl-371^T^ and CACua-24 cultures, the amount of extract obtained was as follows: EHexT = 5.4 mg; EHexC 6.4 mg; EDCMT 958.1 mg; EDCMC = 1,734 mg; EEtOAcT = 6.5 mg; EEtOAcC = 5.1 mg; EMeOHT = 24,102.3 mg; EMeOHC = 15,518.4 mg. Then, using a disc diffusion test, 100 μg and 200 μg of each extract were probed for antimicrobial activity against *T. terrea* SHS 2008^T^ and representative MDR strains, respectively. The strongest activity was observed in the positive control *T. terrea* SHS 2008^T^ since applying 100 μg of EHexT, EHexC, EDCMT, EEtOAcT, and EEtOAcC exhibited inhibition halos up to 44 mm; the most prominent halo was observed with the hexane extract (Fig 2 and S3 Table). The inhibition of the MDR strains was observed when 200 μg of the extract was used. *A. baumannii* 341 was inhibited by EHexT and EHexC, showing halos of 11 and 15 mm, respectively (Fig 2 and S3 Table); *K. pneumoniae* 903137 was inhibited by EHexT (18 mm) and slightly by EHexC (9 mm) (Fig 2 and S3 Table); *E. coli* 3 (9 mm each) and *S. aureus* 4 (16 and 14 mm, respectively) were inhibited by hexane extracts from both TAtl-371^T^ and CACua-24 (Fig 2 and S3 Table), while *P. aeruginosa* strains were not inhibited by any tested extracts (S3 Table). Extracts from DCM, EtOAc, and MeOH inhibited the growth of a few strains (S3 Table). Due to EHexT and EHexC showing the best activity against the MDR strains (Fig 2 and S3 Table), these extracts were selected for further identification of putative antimicrobial metabolites.

### Chromatographic profile and antimicrobial activity by bioautography

**Fig 3:**
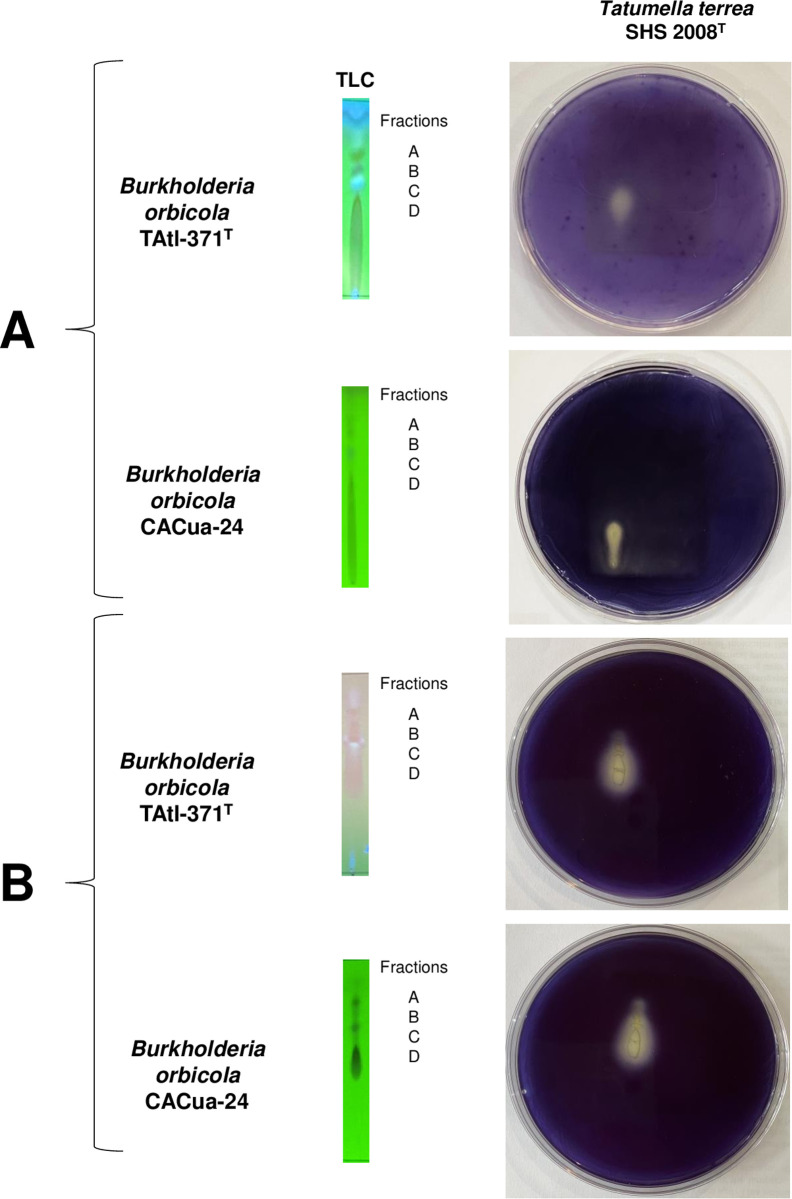
Thin-layer chromatography and bioautography analysis of *Burkholderia orbicola* TAtl-371^T^ and CACua24 hexane extracts. A: corresponds to mobile phase Hex:EtOAc (1:1 v/v). B: corresponds to Hex:DCM:MeOH (3:10:2 v/v). TLC was visualized by UV detection at 302 nm.

The TLC analysis of EHexT and EHexC, using Hex:EtOAc (1:1 v/v) as the mobile phase, showed four spots with UV detection at 302 nm, labeled A-D. An interesting tailing spot was observed at the bottom of the TLC plates (EHexT tailing spot: Rf values from 0 to 3.03; EHexC tailing spot: Rf values from 0 to 1.87) (Fig 3A). The bioautography method was implemented to identify which spots were bioactive against the sensitive strain *T. terrea* SHS 2008^T^. The TLC was covered with soft agar inoculated with the sensitive strain, followed by a second layer of soft agar with MTT. The results revealed that the tailing spot was the only zone of the TLC where *T. terrea* SHS 2008^T^ did not reduce MTT (no purple color was observed) (Fig. 3A), indicating that no living bacteria were present, which suggests antimicrobial activity. A second TLC analysis used a mobile phase of Hex:DCM:MeOH (3:10:2 v/v) (Fig 3B). The tailing spot showed different Rf values (EHexT tailing spot Rf values from 1.7 to 3.58) (EHexC tailing spot Rf values from 1.78 to 2.48). Still, it retained inhibitory activity on *T. terrea* SHS 2008^T^ (Fig 3B). These results confirmed the antimicrobial activity of the metabolites present in the tailing spot. When the tailing spot was exposed to the Salkowski reagent, it turned yellow, indicating the absence of indole derivatives. However, the TLC plates revealed a ninhydrin-positive reaction on the tailing spot, suggesting the putative presence of amide or amine groups.

### Chemical composition analysis of active fraction D

**Fig 4:**
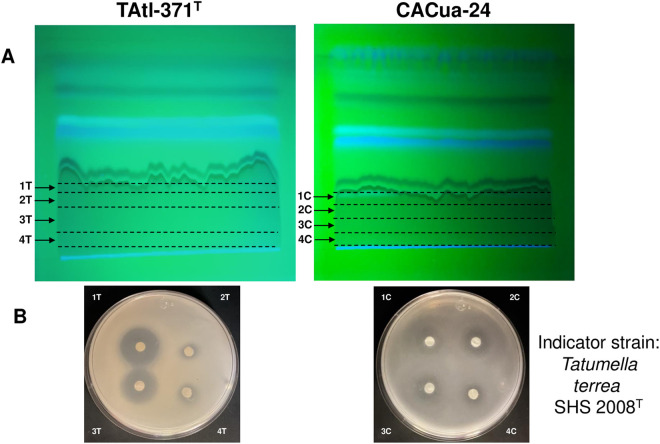
Active hexane extracts from *Burkholderia orbicola* TAtl-371^T^ and CACua-24 cultures were observed by preparative thin-layer chromatography (TLC). A: the mobile phase was Hex:EtOAc (1:1 v/v), and the TLC was visualized with UV light at 302 nm. B: antimicrobial activity of obtained subfractions against *Tatumella terrea* SHS 2008^T^ using the double-layer agar technique. Subfractions from TAtl-371^T^ correspond to 1T, 2T, 3T, and 4T. Subfractions from CACua-24 correspond to 1C, 2C, 3C, and 4C.

Since the tailing spot was identified as the zone with antimicrobial activity, 15 mg of the EHexT and EHexC were separated by preparative TLC to obtain enough samples for further experiments. The tailing spot in the glass TLC was divided into four subfractions (labeled as 1T-4T and 1C-4C) (Fig 4A), scraped, and tested for antimicrobial activity. Subfractions 1T to 3T and 1C to 3C were the most active against *T. terrea* SHS 2008^T^ (Fig 4B). These fractions from each extract (EHexT and EHexC) were mixed and named fraction D. We decided to perform further experiments only with fraction D of EHexT since the antagonism of *B. orbicola* TAtl-371^T^ is the best characterized in the species [7].

A comparison of the ^1^H and ^13^C NMR spectra (S2 and S3 Figs) for fraction D of EHexT with the literature enabled the identification of fragin, a previously reported siderophore produced by *B. cenocepacia* H111 [15]. Fragin was identified from its respective ^1^H NMR signals at δ_H_ 10.06 (s), 5.76 (s), 4.20 (td, *J* = 9.2, 3.0 Hz), 3.86 (ddd, *J* = 14.4, 6.0, 3.1 Hz), 3.59 (ddd, *J *= 14.6, 13.7, 9.0 Hz), 2.24–2.17 (m), 2.14 (td, *J* = 7.4, 2.3 Hz), 1.61–1.55 (m), 1.31–1.23 (m), 1.07 (d, *J* = 6.8 Hz), 0.91 (d, *J* = 6.7 Hz), 0.87 (t, *J *= 7.0 Hz). The ^13^C NMR spectrum of fraction D showed a series of 13 signals of high intensity belonging to the siderophore fragin, which suggests it is the main compound of fraction D (S3 Fig). The ^1^H NMR spectrum (S2 Fig) of fraction D of EHexT revealed the presence of two methyl signals at δ_H_ 1.07 and 0.91 of the isopropyl group close to diazeniumdiolate functional group and the acyl side chain with characteristic signals of a saturated hydrocarbon chain (δ_H_ 1.30–1.23) and methyl group (δ_H_ 0.87, t). HSQC spectra (S4 Fig) further confirmed the identification of fragin in fraction D of EHexT. The mass spectrum (S5 Fig) of fraction D showed the sodium adduct ion peak [M + Na]^+^ at m/z 296.1920 (calcd. for C_13_H_27_N_3_ NaO_3_^+^, 296.1950), indicating the presence of fragin (C_13_H_27_N_3_O_3_). When EHexT was heated with phenol and H_2_SO_4,_ it produced a red color, indicating the presence of the diazeniumdiolate functional group, a characteristic group of fragin [14].

### Fragin-containing fraction D inhibitory spectrum

**Fig 5:**
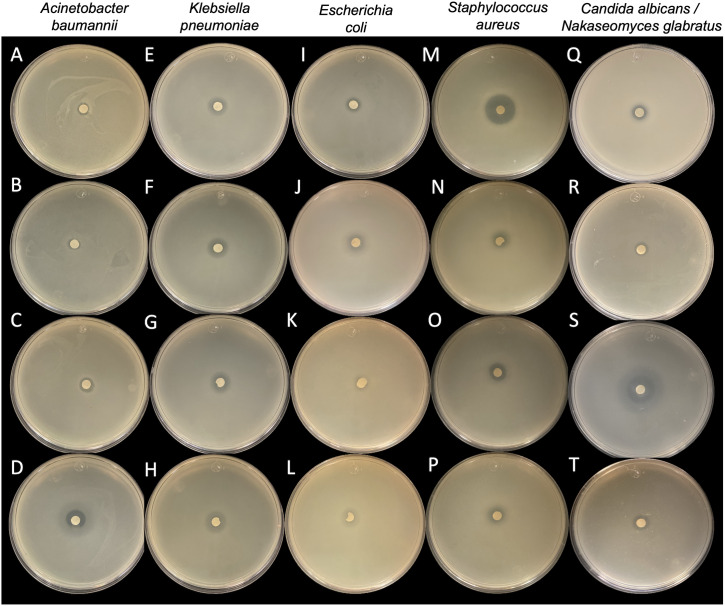
Antimicrobial activity of fragin-containing fraction D from EHexT using the double-layer agar technique. *Acinetobacter baumannii* strains A: 324, B: 345, C: 344, D: 341. *Klebsiella pneumoniae* strains E: 81739, F: 97833, G: 903137, H: 906667. *Escherichia coli* strains I: 1, J: 2, K: 3, L: 4. *Staphylococcus aureus* strains M: 1, N: 2, O: 3, P: 4. *Candida albicans* strains Q: ATCC 10231, R: 30. *Nakaseomyces glabratus* strains S: CBS 138, T: 43.

The inhibitory activity of the fragin-containing fraction D from EHexT was observed against all MDR bacteria tested at 200 μg (Fig 5). Previously, the antifungal activity against *Candida* strains by strains TAtl-371^T^ and CACua-24 was determined [7,8], but the antimicrobial agents were not identified; then, we decided to test the activity of the fragin-containing fraction D against *C. glabrata* (now *N. glabratus*) and *C. albicans* strains to identify the spectrum of this fraction. The results showed an inhibition of both *Candida* and *Nakaseomyces* species (Fig 5).

### Minimum inhibitory concentration of the fragin-containing fraction D

The MIC values of the fragin-containing fraction D from EHexT were determined in selected MDR bacterial and yeast strains (Table 1). The results indicated that *A. baumannii* 351 was inhibited at a concentration of 1024 μg/mL, *S. aureus* 4 at 512 μg/mL, *E. coli* 3 at 256 μg/mL, *K. pneumoniae* 903137, *C. albicans* ATCC 10231, *N. glabratus* CBS138, *S. aureus* ATCC 25923, and *T. terrea* SHS 2008^T^ at 128 μg/mL. Growth was observed in the positive control (bacterial strain in MH medium), and no growth in the negative control (fragin-containing fraction D in MH medium without bacteria). No inhibitory effect was observed in *P. aeruginosa* 11P and 17P.

**Table 1:** Minimum inhibitory concentration (MIC) of fragin-containing fraction D EHexT against selected multidrug-resistant bacteria and yeast.

Microorganisms	MIC (μg/mL)
*Acinetobacter baumannii* 351	1024 ± 0
*Staphylococcus aureus* 4	512 ± 0
*Escherichia coli* 3	256 ± 0
*Klebsiella pneumoniae* 903137	128 ± 0
*S. aureus* ATCC 25923	128 ± 0
*Tatumella terrea* SHS 2008^T^	128 ± 0
*Candida albicans* ATCC 10231	128 ± 0
*Nakaseomyces glabrata* CBS138	128 ± 0
*Pseudomonas aeruginosa* 11P	No inhibition
*P. aeruginosa* 17P	No inhibition

### Fragin-containing fraction D toxicity in the *Galleria mellonella* model

**Fig 6:**
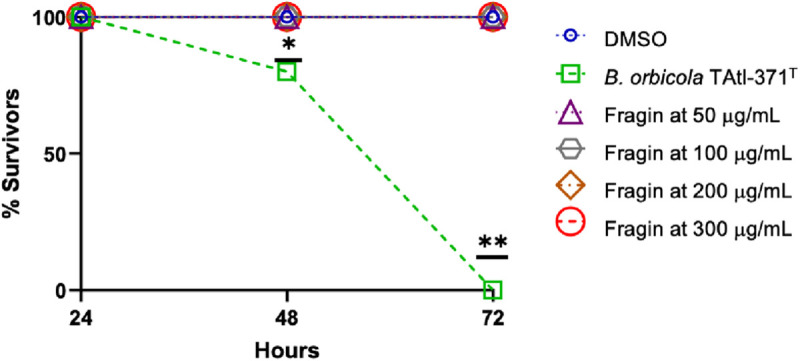
Survival percentage of *Galleria mellonella* larvae exposed to fragin-containing fraction D. The experiment was performed in triplicate. The statistical analysis was performed using a two-way ANOVA test; the differences are denoted by one or two asterisks. *p* = 0.05.

The analysis of the fragin-containing fraction D tested on *G. mellonella* showed no harmful effect on the larvae at any of the analyzed concentrations (50, 100, 200, and 300 μg/mL) (Fig 6). The inoculation with *B. orbicola* TAtl-371^T^ killed 100% of the larvae at 72 h.

### Fragin biosynthetic genes

The organization of fragin biosynthetic clusters in strains TAtl-371^T^ and CACua-24 (Fig S6) was identical to that of the *ham* genes described in *Burkholderia cenocepacia* H111 [15], with more than 99.0% identity. The *ham* genes were also found in many *B. cenocepacia* strains, as well as in a few *B. cepacia*, *B. stabilis,* and *Paraburkholderia phenazinium* strains.

## Discussion

The antimicrobial activity of *Burkholderia* species has been noted in recent years, with numerous new compounds recently described [5,6]. One of the most recent antimicrobial compounds elucidated in *Burkholderia* is thailandene, produced by *Burkholderia thailandensis* DW503. Variants A and B inhibit *Bacillus subtilis*, *S. aureus,* and *Saccharomyces cerevisiae* [21], demonstrating the ability of *Burkholderia* to produce compounds with activity against various microorganisms, including bacteria and yeasts.

In the current study, we evaluated the ability of two *B. orbicola* strains to inhibit MDR bacteria on PDA using the double-layer agar technique. Previously, we determined that *B. orbicola* TAtl-371^T^ has a broad antimicrobial spectrum, inhibiting many strains from the Bcc, as well as other genera such as *Paraburkholderia*, *Cupriavidus*, *Escherichia*, *Salmonella*, phytopathogenic bacteria, fungi and oomycetes, and clinically relevant yeast species [7]. The previous antimicrobial spectrum of the strain CACua-24 includes only some strains of fungi, yeasts, oomycetes, and bacteria [8,10]. In the present work, *B. orbicola* TAtl-371^T^ and CACua-24 were found to inhibit several MDR strains of *A. baumannii*, *K. pneumoniae, P. aeruginosa, E. coli*, and *S. aureus*, thereby increasing their antimicrobial spectrum and potential as a source of antimicrobial compounds. Sequential extraction of metabolites from PDA cultures of *B. orbicola* using Hex, DCM, EtOAc, and MeOH showed that Hex was the best solvent to extract antimicrobials from agar cultures of both strains. Many antimicrobial compounds produced by *Burkholderia* have been commonly isolated with MeOH, EtOAc, and DCM [7,22–29]. This is the first work where hexane extracts from agar cultures of *Burkholderia* strains convey antimicrobial activity.

The Hex extracts from both *B. orbicola* strains (EHexT and EHexC) exhibited the best inhibition of MDR strains compared to other solvent extracts, so these extracts were selected for further characterization. The TLC analysis of EHexT and EHexC showed an interesting tailing spot at the bottom of the plates. Using the bioautography method, we determined that this tailing spot had activity against *T. terrea* SHS 2008^T^, a strain highly sensitive to the antimicrobials produced by *B. orbicola* [7]. The tailing phenomenon on TLC plates is commonly attributed to high sample concentration [30] or high sample pH [31]. To diminish the tailing effect in this spot and determine if there was more than one compound, three variations of TLC were applied: different solvent systems as mobile phases, different Hex extract amounts on the plates, and the addition of formic acid to reduce the pH of the samples, a method reported to compact tailing effects [31]. In all cases, this spot on the TLC always revealed a similar tailing effect. When using different solvent systems, the tailing spot exhibited different R_f_ values but conserved its antimicrobial activity. Since indole derivatives, such as indole pyruvic acid found in plant-associated bacteria, were previously detectable as a tailing spot [20], the TLC plates of EHexT and EHexC were exposed to the Salkowski reagent, commonly used to identify indole derivatives from bacteria. Still, the tailing spot turned yellow, indicating the absence of indole derivatives. However, this fraction turned pink when ninhydrin was applied to the TLC plates. This is a common reaction when compounds containing amide and primary/secondary amine groups are present [32,33], suggesting the presence of compounds containing these functional groups.

The next step in the study was to determine the compounds in the tailing spot by spectroscopic analysis. The tailing spot subfractions with antimicrobial activity were mixed and analyzed as fraction D, which showed a majoritarian compound identified as fragin, a previously reported siderophore with inhibitory activity against Gram-positive and -negative bacteria [16], suggesting that it could be related to the antibacterial activity of Hex extracts of *B. orbicola* against *T. terrea* SHS 2008^T^, *A. baumannii* 341, *K. pneumoniae* 903137, and *S. aureus* 4. The identification of fragin aligns with our previous suggestion that *B. orbicola* TAtl-371^T^ produces antimicrobial siderophores on PDA media [7]. The presence of fragin on EHexT was confirmed when samples were heated with phenol and H_2_SO_4,_ and a red color was observed, which suggests the presence of compounds containing the diazeniumdiolate group [14]. This group is present in C-diazeniumdiolates siderophores produced by plant-associated bacteria [34] and in *B. cenocepacia* H111, which is crucial for the antimicrobial activity of the siderophore fragin [15,16].

Initially, fragin was isolated by growing a *Pseudomonas* sp. strain in a medium containing 0.02% K_2_HPO_4_, 0.05% peptone, 0.02% yeast extract, and 1% glucose for ten days at 28°C and extracted with benzene [14]. In the second study, Jenul et al. [15] grew *B. cenocepacia* H111 in the ABG minimal medium for 72 h at 37°C and extracted it with chloroform. In the current study, we identified fragin in the hexane extract of *B. orbicola* TAtl-371^T^ grown in PDA medium for 72 h at 30°C. The presence of fragin in the Hex extract is notable, as its identification and isolation have been achieved from extracts with significant polarity.

Fragin was first isolated in a screening for bacterial plant growth inhibitors in 1967, finding *Pseudomonas* among them [14]. Later, the compound was purified from *Burkholderia cenocepacia* H111, described as a diazeniumdiolate NRP synthesized by a set of 7 genes [15] with activity against the fungi *Fusarium solani* and the Gram-positive bacteria *Bacillus cereus*, *B. subtilis*, *Bacillus thuringiensis,* and *S. aureus* [15,16]. The action of this molecule on Gram-negative bacteria was weak, affecting only *E. coli* [16]. Our results suggest that fragin could inhibit other Gram-negative species, such as *A. baumannii* and *K. pneumoniae*. Even though the antibacterial activity of fragin against Gram-positive, such as *S. aureus, B. cereus*, *B. subtilis,* and *B. thuringensis* was previously reported [16], as far as we know, this is the first time that a diazeniumdiolate siderophore is identified in organic extracts from bacterial cultures with activity against MDR strains of *A. baumannii* and *K. pneumoniae.* Only a few compounds with activity against *A. baumannii* are known on the Bcc strains, including enacyloxin IIa in *B. ambifaria* [35] and gladiolin in *B. gladioli* [28]. Although *K. pneumoniae* is inhibited by two siderophores produced by *Burkholderia* [5], none belong to the diazeniumdiolate siderophore class.

Additionally, we reported the antifungal activity of *B. orbicola* strains against *N. glabratus* and *C. albicans* [7,8], and observed a relationship between siderophore production and the inhibition of *N. glabratus* [7]. However, no antifungal metabolites were identified at that time. Since fragin showed activity against fungal growth [15], we decided to test the siderophore fragin-containing fraction D against *N. glabratus* and *C. albicans*. The fraction D showed inhibition of strains from both species, suggesting a strong relationship with the antagonism of *B.* orbicola against yeast. Compounds produced by *Burkholderia* with activity against yeasts are known [5], but none have been reported as siderophores.

The genetic organization of fragin genes found on *B. orbicola* TAtl-371^T^ and CACua-24 was identical to the report in *B. cenocepacia* H111 [15], which proposes both species as potential producers of fragin as an antimicrobial metabolite. This is expected as *B. orbicola* is the closest species to *B. cenocepacia* on the Bcc.

## Conclusion

The rapid development of resistance, the common presence of MDR among clinical isolates, and the severity of infections caused by *A. baumannii*, *S. aureus*, and *K. pneumoniae* are global concerns that prompt the scientific community to seek new therapeutic alternatives for treating these pathogens. The capacity of *B. orbicola* to produce fragin and its potential activity against MDR bacterial strains of *A. baumannii*, *S. aureus*, and *K. pneumoniae* is a promising alternative for treating infections caused by these pathogens. Fragin and similar molecules were previously suggested as a promising source for drug development. Our results support this idea and suggest valuable potential for these molecules to be used in combating health problems caused by MDR strains. Moreover, *B. orbicola* warrants further study to elucidate the molecules that inhibit *P. aeruginosa,* given that fragin is unable to impede its growth.

## Supporting information

S1 Fig Antibacterial activity of *Burkholderia orbicola* TAtl-371^T^ and CACua-24 against *Tatumella terrea* SHS 2008^T^ and multidrug-resistant bacteria from the species *Acinetobacter baumannii*, *Klebsiella pneumoniae*, *Pseudomonas aeruginosa*, *Escherichia coli* and *Staphylococcus aureus* by double-layer agar technique. (PDF)

S2 Fig ^1^H NMR spectrum of fraction D (600 MHz, CDCl_3_). (PDF)

S3 Fig ^13^C NMR spectrum of fraction D (150 MHz, CDCl_3_). (PDF)

S4 Fig HSQC spectrum of fraction D (600 MHz, CDCl_3_). (PDF)

S5 Fig Mass spectrum (positive ion mode) of fraction D. (PDF)

S6 Fig Fragin Ham biosynthesis cluster present in some *Burkholderia orbicola* strains. HamE, polyketide cyclase/dehydrase. HamD, consists of an adenylation domain, thiolation domain and reductase domain. HamC, p-aminobenzoate N-oxygenase AurF. HamB, RmlC-like cupin domain superfamily. HamA, Haem-oxygenase-like, multi-helical. HamF, condensation domain. HamG, aminotransferase class-III. (PDF)

S1 Table Antimicrobial susceptibility of multidrug-resistant bacteria. (DOCX)

S2 Table Inhibitory activity of *Burkholderia orbicola* TAtl-371^T^ and CACua-24 on multidrug-resistant bacteria by double-layer agar technique. (DOCX)

S3 Table Inhibitory activity of *Burkholderia orbicola* TAtl-371^T^ extracts and CACua-24 on multidrug-resistant bacteria. (DOCX)
