# Supplementary material for: Dissecting the inhibitory activity of Burkholderia orbicola against Gram-positive and - negative multidrug-resistant bacteria
Source: PLoS One. 2025 Jun 30;20(6):e0326906. doi: 10.1371/journal.pone.0326906 (PMC12208415; doi:10.1371/journal.pone.0326906)
Supplement: S1 Fig — (PDF) [file pone.0326906.s001.pdf]

S1 Figure.

Antibacterial activity of *Burkholderia orbicola* TAtl-371^{T} and CACua-24 against *Tatumella terrea* SHS 2008^{T} and multidrug-resistant bacteria from the species *Acinetobacter baumannii*, *Klebsiella pneumoniae*, *Pseudomonas aeruginosa*, *Escherichia coli* and *Staphylococcus aureus* by double-layer agar technique.

Burkholderia orbicola vs *Acinetobacter baumannii*

TAtl-371^{T}

CACua-24

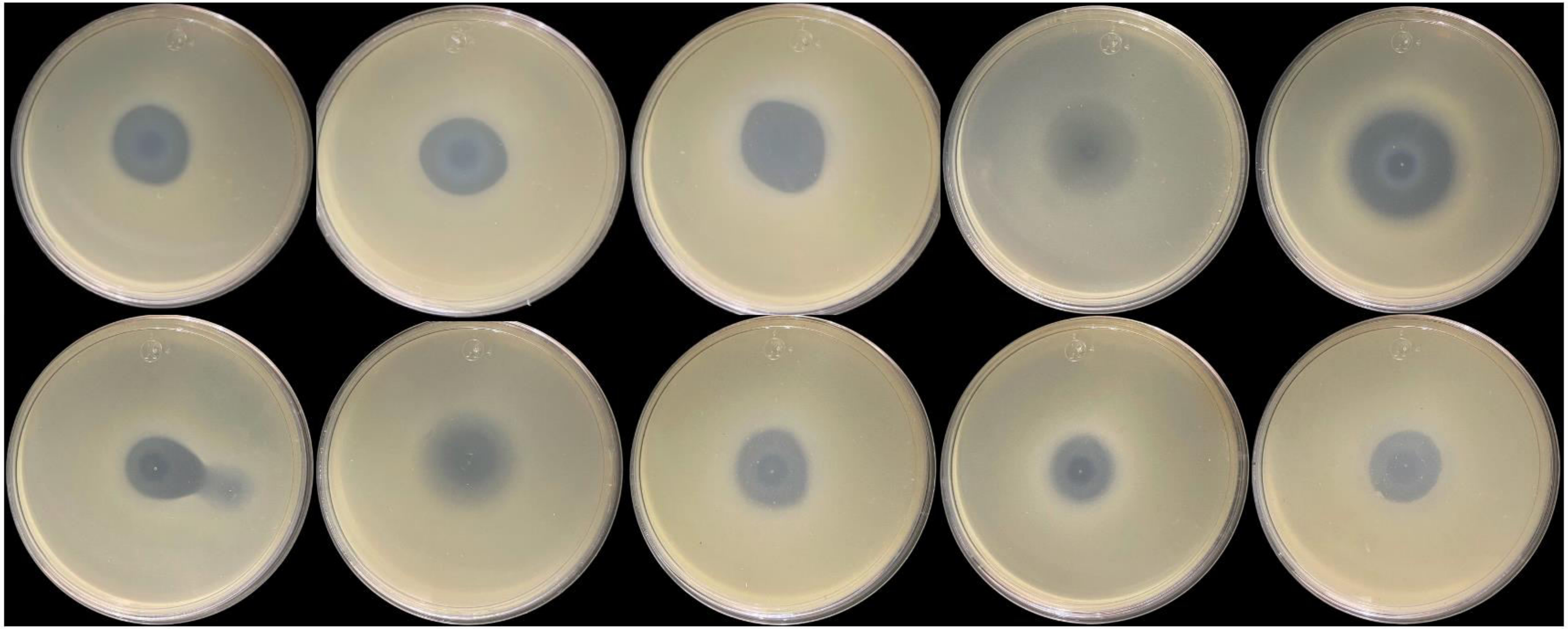

256 324 341 344 345

Burkholderia orbicola vs *Klebsiella pneumoniae*

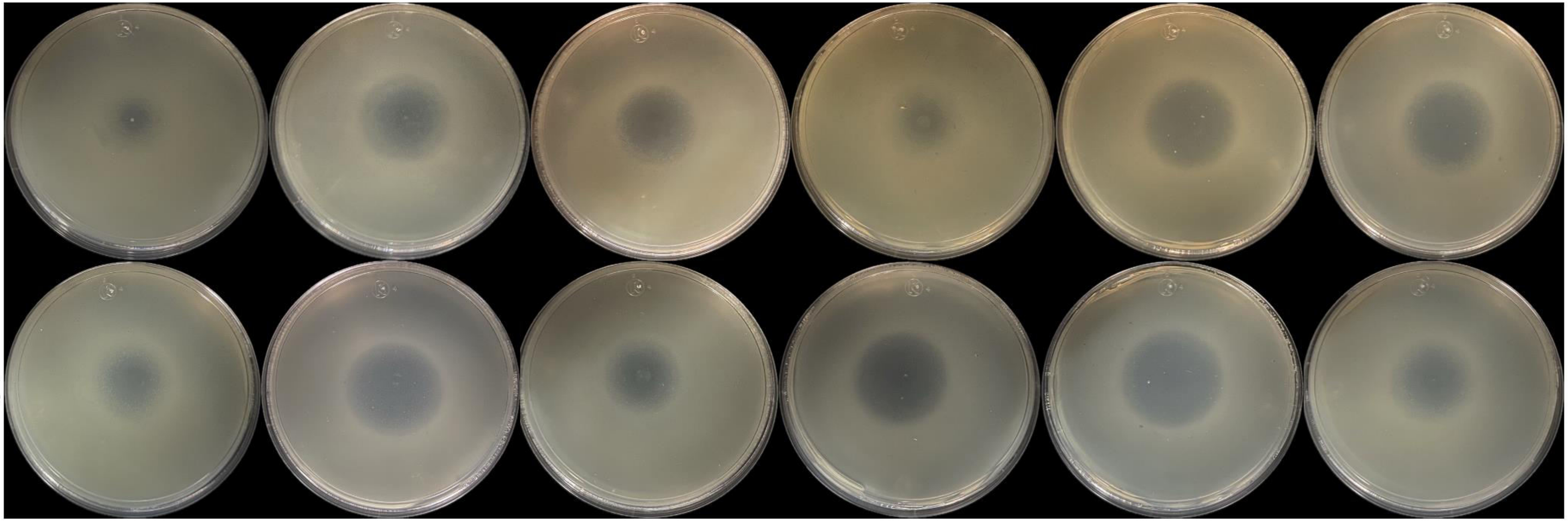

Burkholderia orbicola vs *Pseudomonas aeruginosa*

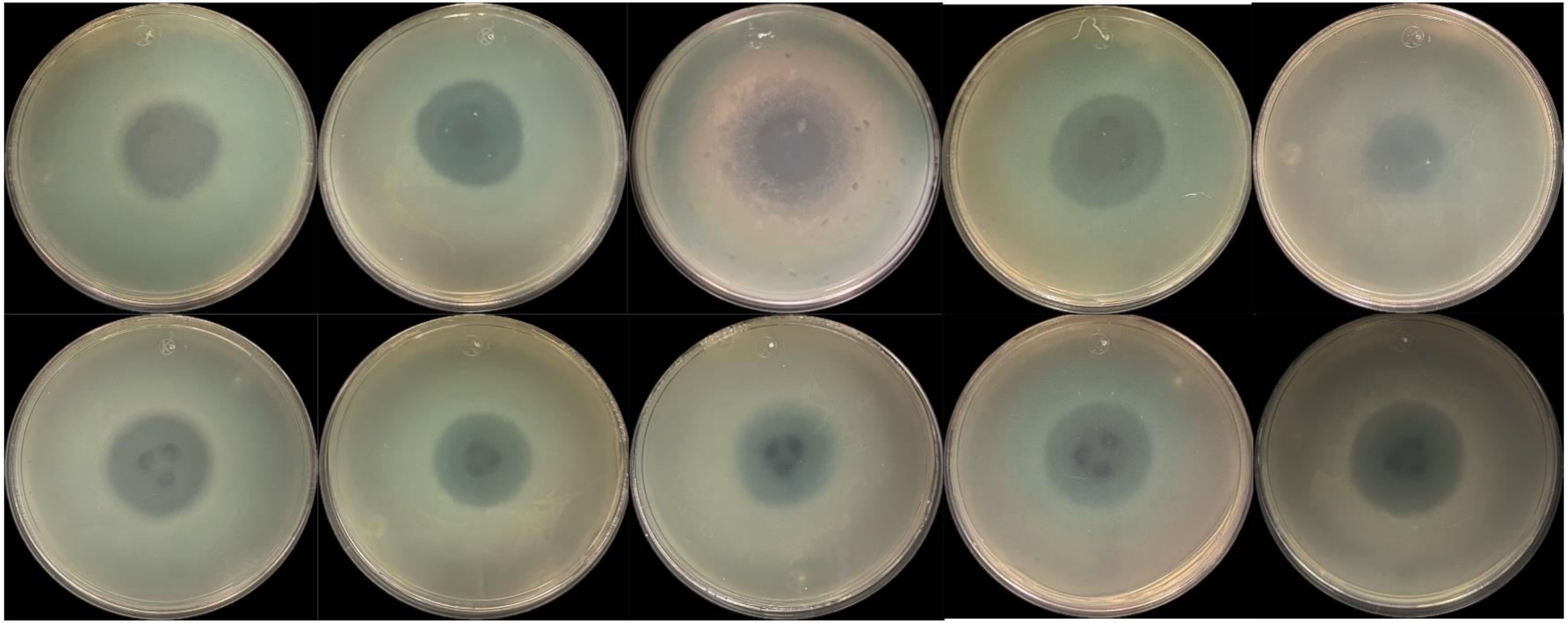

TAtl-371^{T}

CACua-24

1P 2P 4P 11P 12P

Burkholderia orbicola vs *Pseudomonas aeruginosa*

TAtl-371^{T}

CACua-24

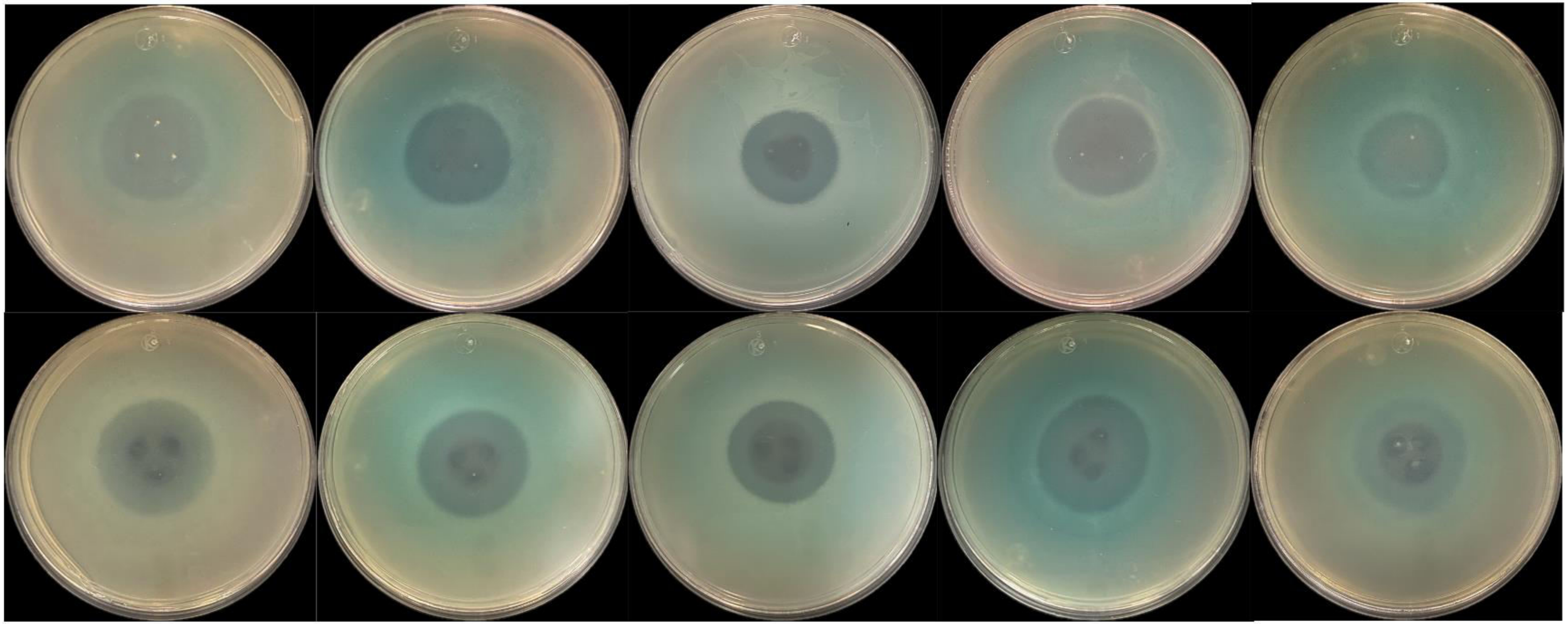

16P 17P 22P 26P 30P

Burkholderia orbicola vs *Escherichia coli*

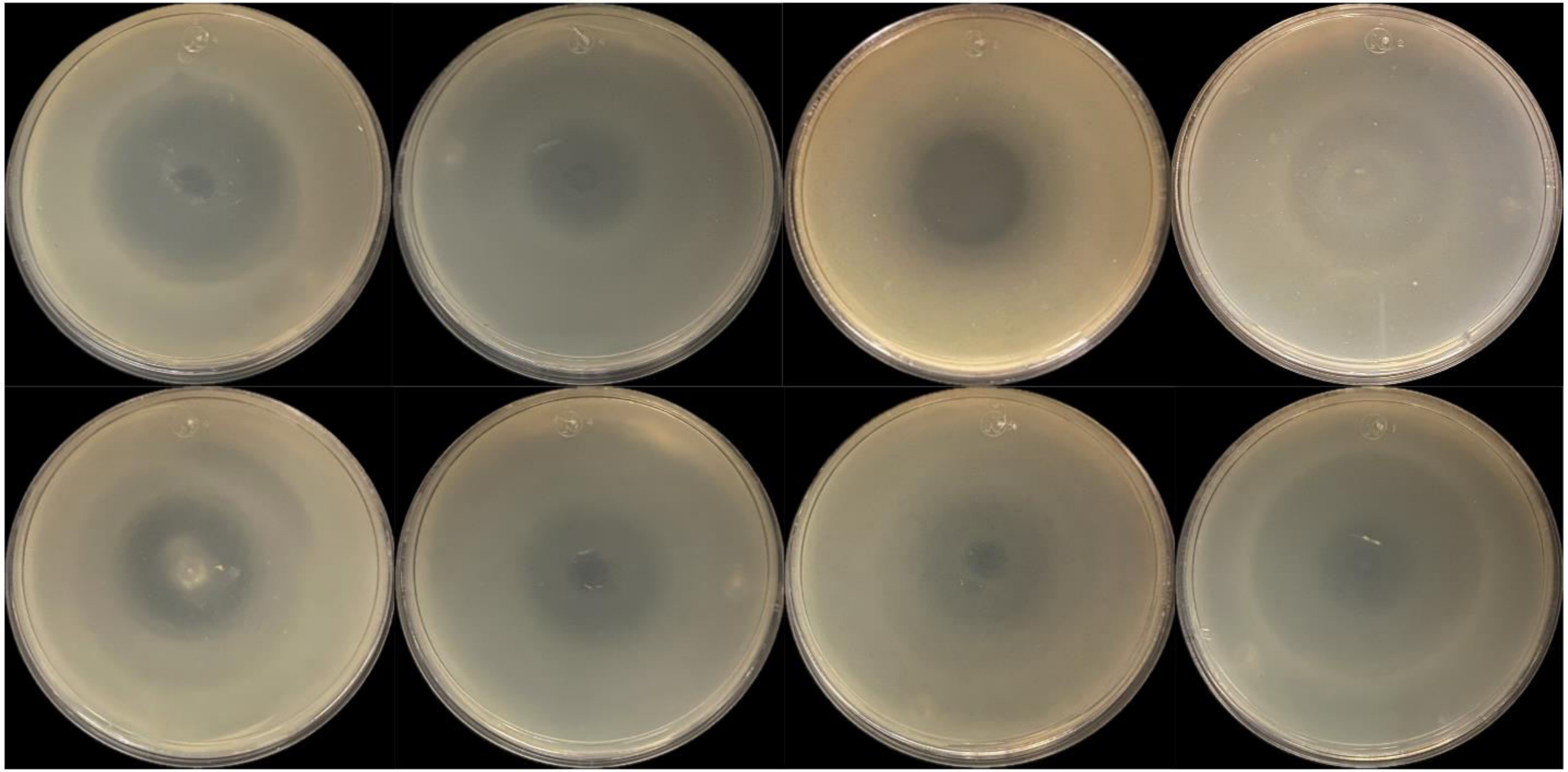

TAtl-371^T

CACua-24

1 2 3 4

Burkholderia orbicola vs *Staphylococcus aureus*

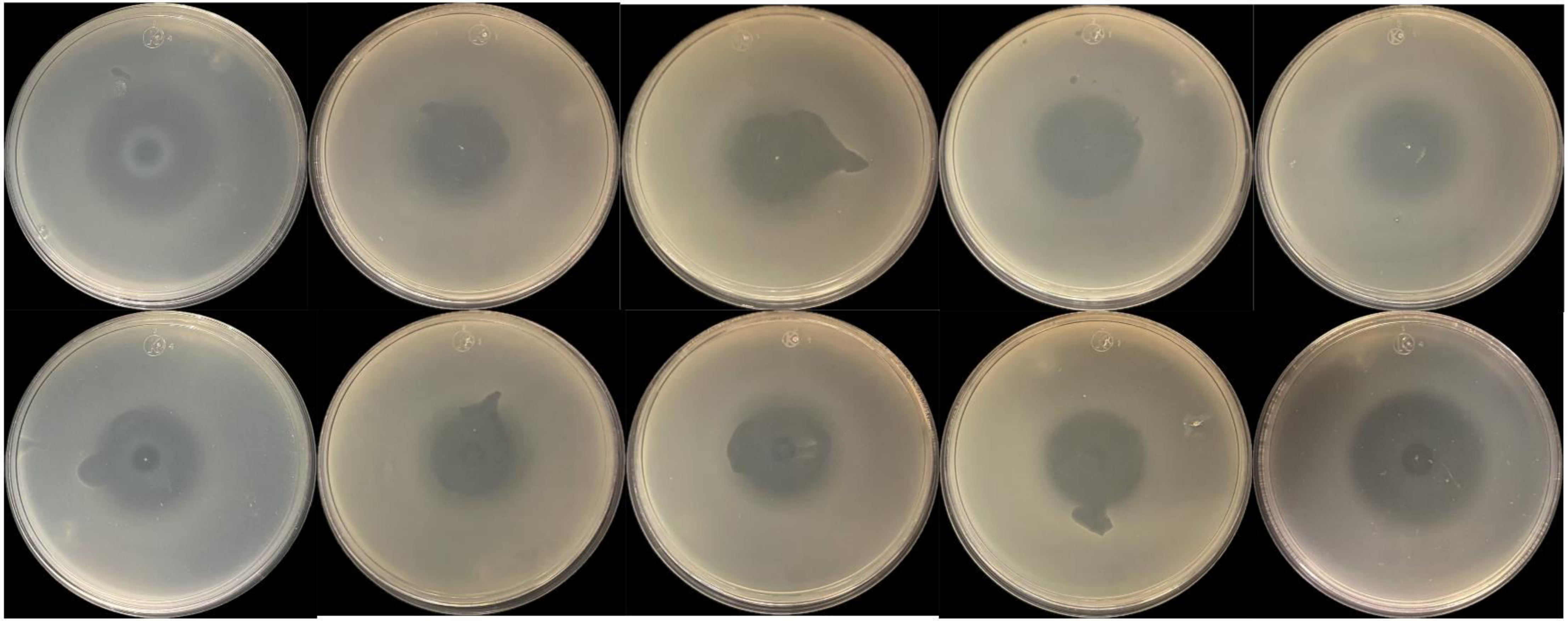

TAtl-371^{T}

CACua-24

ATCC 25923 | 1 | 2 | 3 | 4
