# Supplementary figures and images for: Dissecting the inhibitory activity of Burkholderia orbicola against Gram-positive and - negative multidrug-resistant bacteria
Source: PLoS One. 2025 Jun 30;20(6):e0326906. doi: 10.1371/journal.pone.0326906 (PMC12208415; doi:10.1371/journal.pone.0326906)

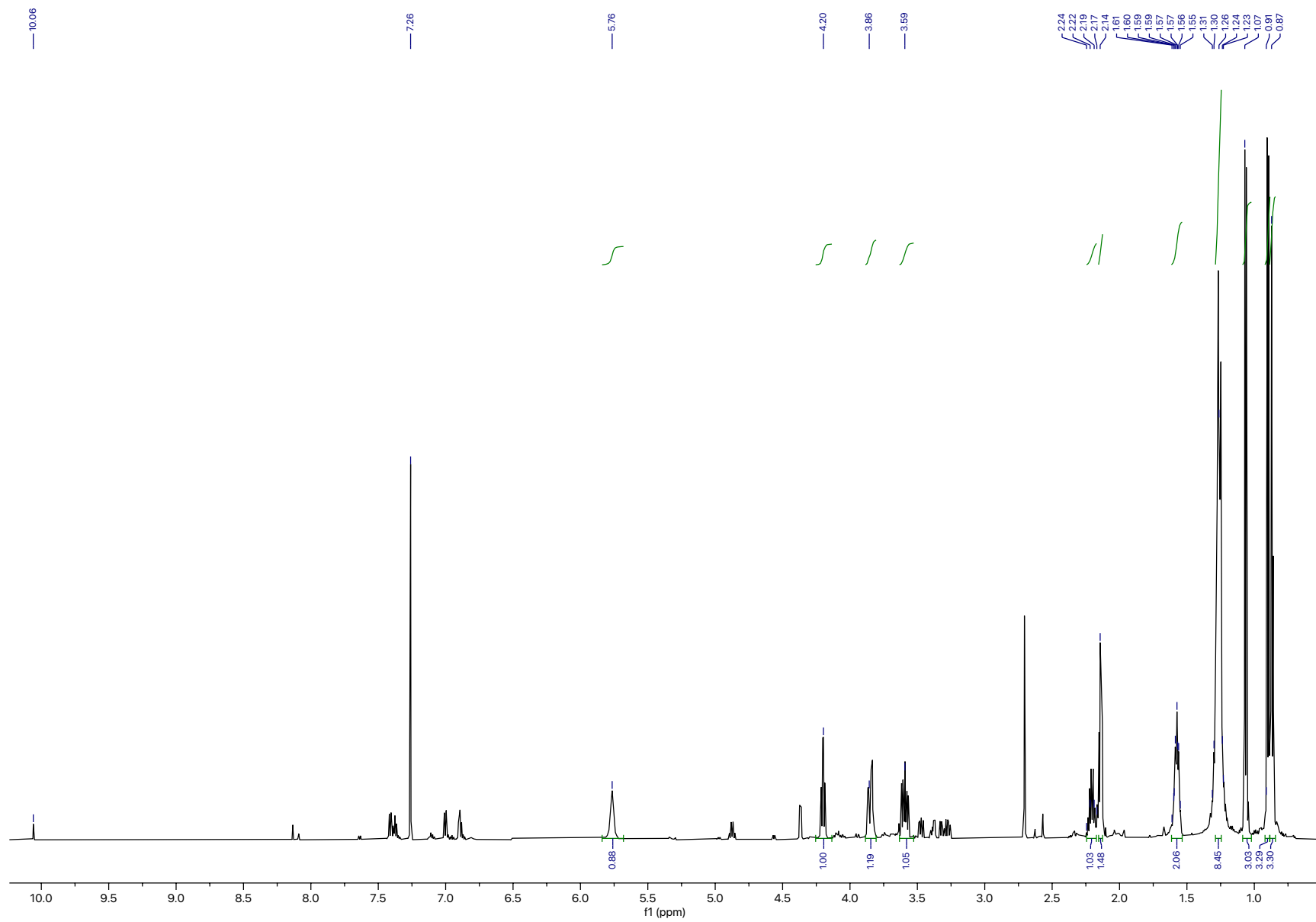

**S2 Figure.** <sup>1</sup>H NMR spectrum of fraction D (600 MHz, CDCl<sub>3</sub>).

Supplement: S2 Fig — (PDF) [file pone.0326906.s002.pdf]

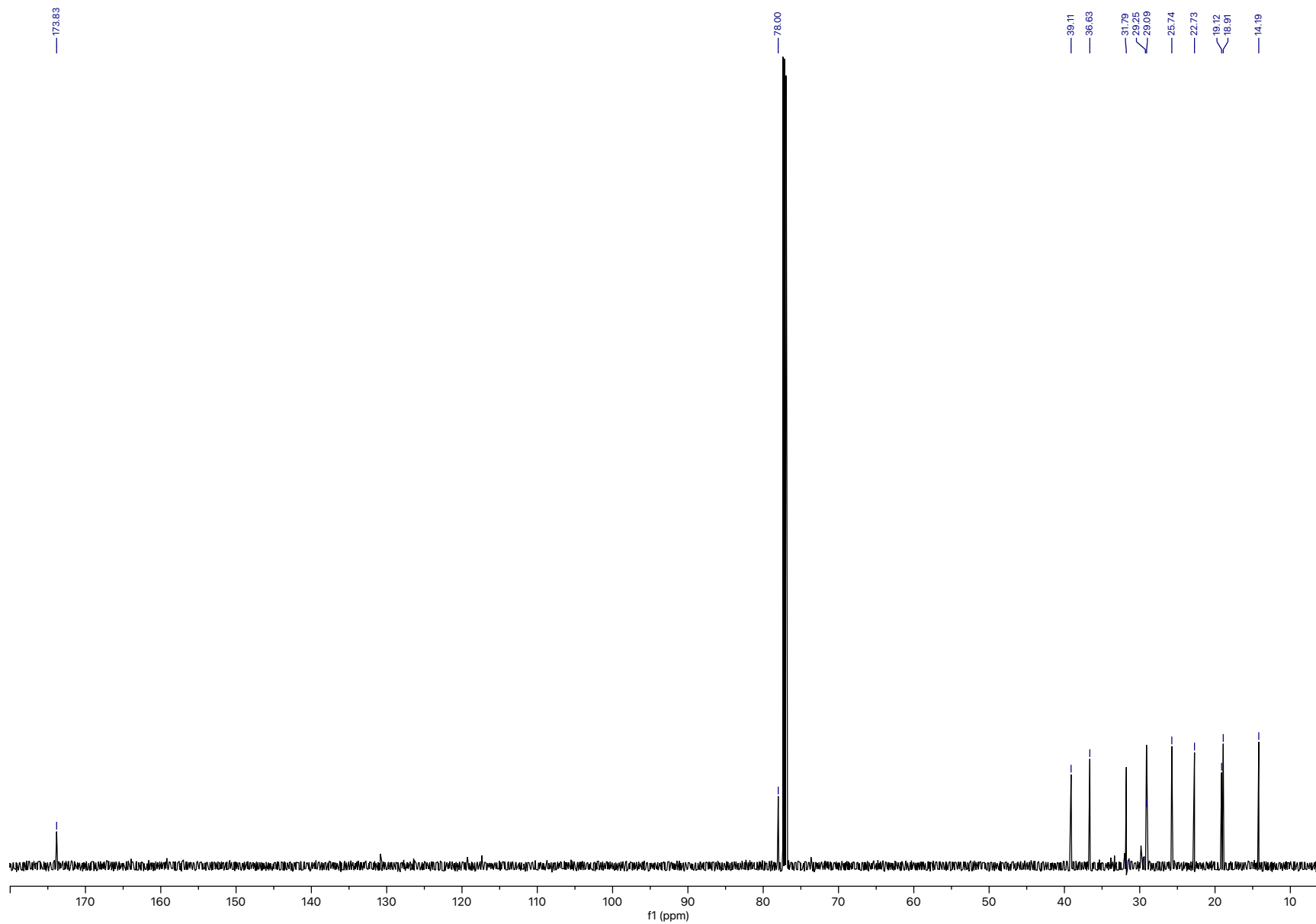

**S3 Figure.** <sup>13</sup>C NMR spectrum of fraction D (150 MHz, CDCl<sub>3</sub>).

Supplement: S3 Fig — (PDF) [file pone.0326906.s003.pdf]

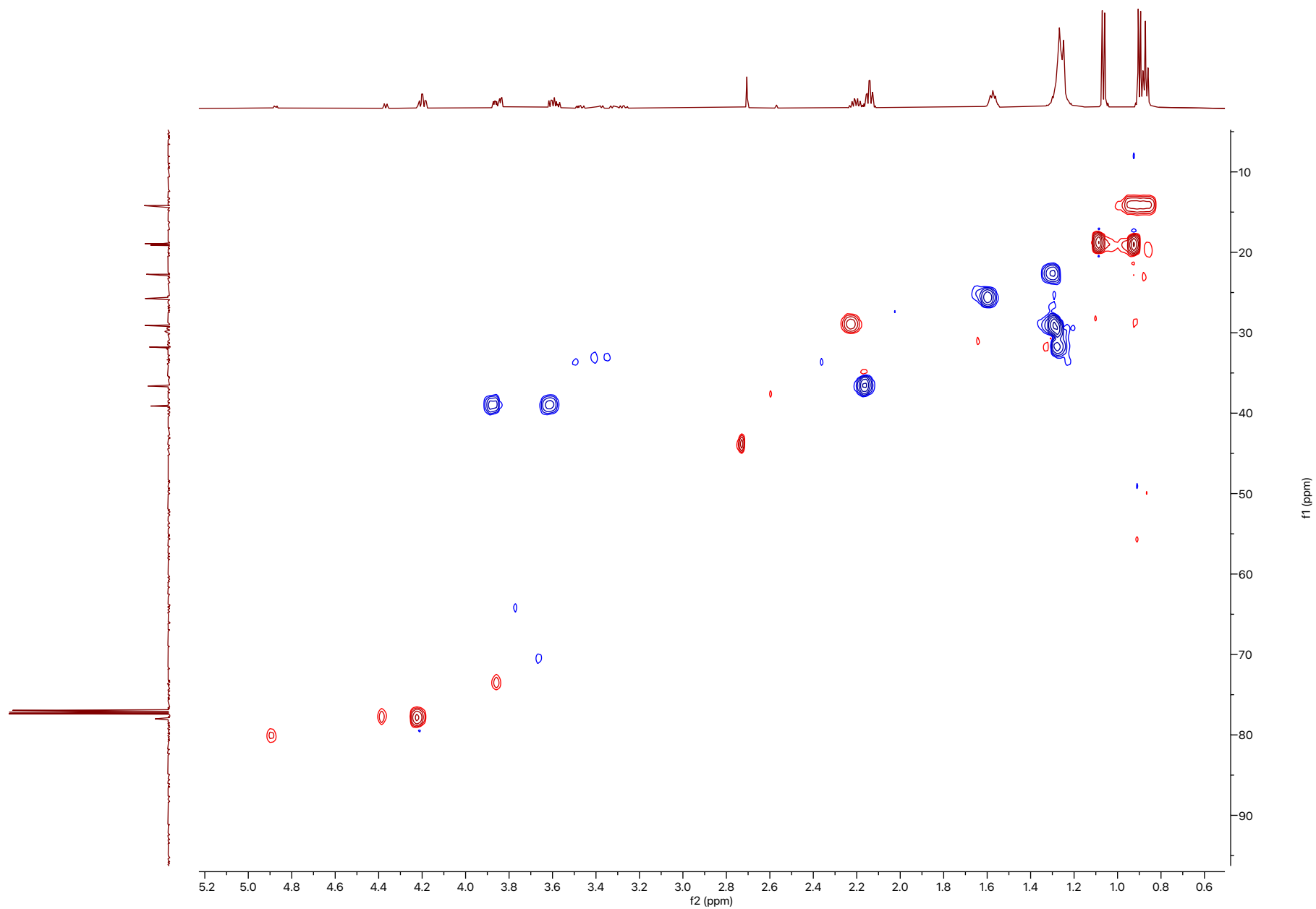

**S4 Figure.** HSQC spectrum of fraction D (600 MHz,  $\text{CDCl}_3$ ).

Supplement: S4 Fig — (PDF) [file pone.0326906.s004.pdf]
