# Supplementary material for: Dissecting the inhibitory activity of Burkholderia orbicola against Gram-positive and - negative multidrug-resistant bacteria
Source: PLoS One. 2025 Jun 30;20(6):e0326906. doi: 10.1371/journal.pone.0326906 (PMC12208415; doi:10.1371/journal.pone.0326906)
Supplement: S6 Fig — (PDF) [file pone.0326906.s006.pdf]

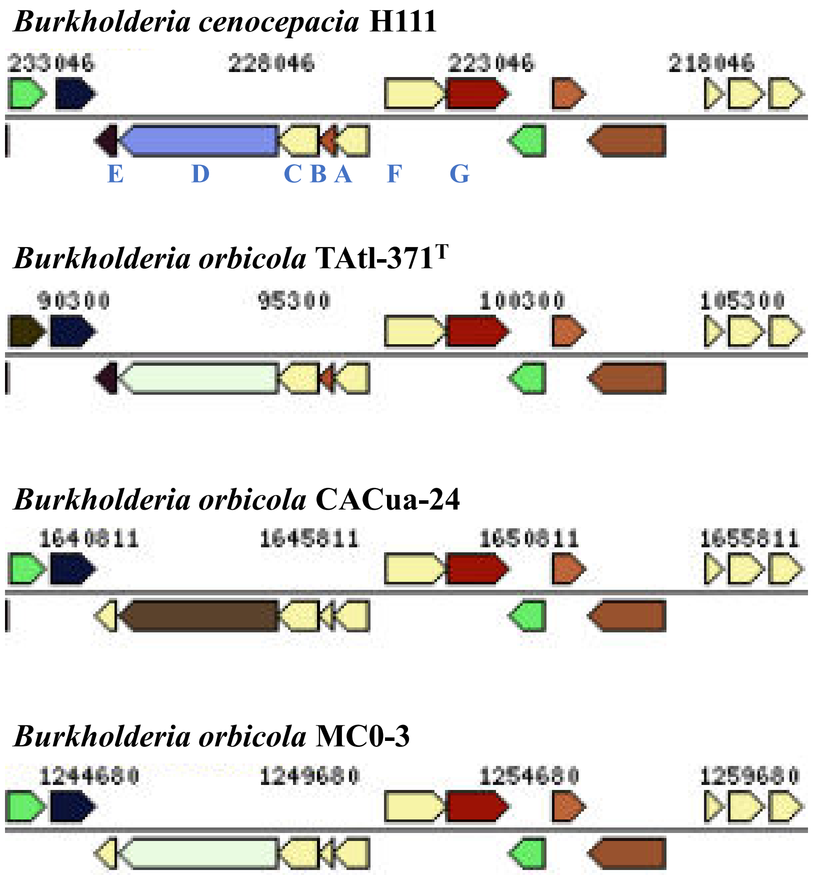

S6 Figure. Fragin Ham biosynthesis cluster present in some *Burkholderia orbicola* strains. HamE, polyketide cyclase/dehydrase. HamD, consists of an adenylation domain, thiolation domain and reductase domain. HamC, p-aminobenzoate N-oxygenase AurF. HamB, RmlC-like cupin domain superfamily. HamA, Haem-oxygenase-like, multi-helical. HamF, condensation domain. HamG, aminotransferase class-III.
