# Supplementary material for: Dissecting the inhibitory activity of Burkholderia orbicola against Gram-positive and - negative multidrug-resistant bacteria
Source: PLoS One. 2025 Jun 30;20(6):e0326906. doi: 10.1371/journal.pone.0326906 (PMC12208415; doi:10.1371/journal.pone.0326906)
Supplement: S1 Table — (DOCX) [file pone.0326906.s007.docx]

| **S1 Table. Antmicrobial susceptibility of multidrug resistant bacteria.** | | | | | | |  |  |  |  |
| --- | --- | --- | --- | --- | --- | --- | --- | --- | --- | --- |
|  | ***Pseudomonas aeruginosa*** | | | | | | | | | |
| Antibiotic/Clinical Isolates | 1P | 2P | 4P | 11P | 12P | 16P | 17P | 22P | 26P | 30P |
| Piperacilin-Tazobactam | S | R | R | I | S | I | R | S | I | I |
| Ceftazidime | S | R | R | R | I | R | R | S | S | S |
| Cefepime | I | I | I | R | S | I | R | I | S | S |
| Doripenem | S | S | S | R | S | S | R | S | R | R |
| Imipenem | S | S | S | R | S | R | R | S | R | R |
| Meropenem | S | S | S | R | S | S | NT | S | R | R |
| Amikacin | I | S | S | R | S | S | S | I | S | S |
| Gentamicin | I | S | S | R | S | S | S | I | S | S |
| Ciprofloxacin | R | S | S | R | R | S | S | S | I | I |
| Tigecycline | R | R | R | R | R | R | NT | R | R | R |
| Colistin | S | S | S | S | S | S | S | S | S | S |
|  |  |  |  |  |  |  |  |  |  |  |
| ***Acinetobacter baumannii*** | | | | | | | | | | |
| Antibiotic/Clinical Isolates | 140 | 194 | 256 | 324 | 341 | 343 | 344 | 345 | 351 |  |
| Tetracycline-Clavulanic acid | S |  | R | R | R | R | R | R | R |  |
| Piperacilin | S |  | R | R | R | R | R | R | R |  |
| Ceftriaxone | I |  | R | R | R | R | R | R | R |  |
| Cefepim | S |  | R | R | R | R | R | R | R |  |
| Meropenem | S |  | R | R | R | R | R | R | R |  |
| Levofloxacin | S |  | R | R | R | I | R | R | I |  |
| Minocycline | S |  | S | R | I | S | R | R | S |  |
| Tetracycline | S |  | R | R | R | R | R | R | R |  |
| Tigecycline | S |  | S | I | R | S | R | R | S |  |
|  |  |  |  |  |  |  |  |  |  |  |
| ***Escherichia coli*** | | | | | | | | | | |
| Antibiotic/Clinical Isolates | 1 | 2 | 3 | 4 |  |  |  |  |  |  |
| Amoxicilin | R | R | R | R |  |  |  |  |  |  |
| Ampicilin | R | R | R | R |  |  |  |  |  |  |
| Ampicilin-Sulbactam | I | R | R | R |  |  |  |  |  |  |
| Cefalotin | R | R | R | R |  |  |  |  |  |  |
| Cefuroxime | R | R | NT | NT |  |  |  |  |  |  |
| Cefotaxime | R | R | R | R |  |  |  |  |  |  |
| Ceftazidime | R | R | R | R |  |  |  |  |  |  |
| Ceftriaxone | R | R | R | R |  |  |  |  |  |  |
| Cefepime | R | R | R | R |  |  |  |  |  |  |
| Ertapenem | S | S | S | S |  |  |  |  |  |  |
| Meropenem | S | S | S | S |  |  |  |  |  |  |
| Amikacin | S | S | S | S |  |  |  |  |  |  |
| Gentamicin | S | R | R | R |  |  |  |  |  |  |
| Ciprofloxacin | S | R | R | R |  |  |  |  |  |  |
| Norfloxacin | S | R | R | R |  |  |  |  |  |  |
| Fosfomycin | S | NT | NT | NT |  |  |  |  |  |  |
| Nitrofurantoin | S | S | S | S |  |  |  |  |  |  |
| Trimethoprim-sulfamethoxazole | R | R | R | R |  |  |  |  |  |  |
|  |  |  |  |  |  |  |  |  |  |  |
| ***Staphylococcus aureus*** | | | | | | | | | | |
| Antibiotic/Clinical Isolates | 1 | 2 | 3 | 4 |  |  |  |  |  |  |
| Ampicilin | NT | NT | NT | NT |  |  |  |  |  |  |
| Oxacilin | R | R | R | R |  |  |  |  |  |  |
| Gentamicin | S | S | S | S |  |  |  |  |  |  |
| Ciprofloxacin | R | R | R | R |  |  |  |  |  |  |
| Levofloxacin | R | R | R | R |  |  |  |  |  |  |
| Moxifloxacin | R | R | R | R |  |  |  |  |  |  |
| Erythromycin | R | R | R | R |  |  |  |  |  |  |
| Clindamycin | R | R | R | R |  |  |  |  |  |  |
| Linezolid | S | S | S | S |  |  |  |  |  |  |
| Daptomycin | S | S | S | S |  |  |  |  |  |  |
| Vancomycin | S | S | S | S |  |  |  |  |  |  |
| Doxicyclin | S | S | S | S |  |  |  |  |  |  |
| Tetracycline | S | S | S | S |  |  |  |  |  |  |
| Tigecycline | S | S | S | S |  |  |  |  |  |  |
| Nitrofurantoin | S | S | S | S |  |  |  |  |  |  |
| Rifampicin | S | S | S | S |  |  |  |  |  |  |
| Trimethoprim-sulfamethoxazole | S | S | S | S |  |  |  |  |  |  |
|  |  |  |  |  |  |  |  |  |  |  |
|  |  |  |  |  |  |  |  |  |  |  |
| ***Klebsiella pneumoniae*** | | | | | | | | | | |
| Antibiotic/Clinical Isolates | 97833 | 9851043 | 81739 | 903137 | 945626 | 906667 |  |  |  |  |
| Amikacin | < = 2 | < = 2 | < = 2 | < = 2 | < = 2 | < = 2 |  |  |  |  |
| Gentamicin | < = 1 | < = 1 | < = 1 | < = 1 | < = 1 | < = 1 |  |  |  |  |
| Cefoxitin | < = 4 | > = 64 | < = 4 | < = 4 | < = 4 | < = 4 |  |  |  |  |
| Ceftriaxone | < = 1 | < = 1 | < = 1 | < = 1 | < = 1 | < = 1 |  |  |  |  |
| Ceftazidime | < = 1 | < = 1 | < = 1 | < = 1 | < = 1 | < = 1 |  |  |  |  |
| Cefepime | < = 1 | < = 1 | < = 1 | < = 1 | < = 1 | < = 1 |  |  |  |  |
| Meropenem | NT | NT | < = 0.25 | NT | < = 0.25 | < = 0.5 |  |  |  |  |
| Ertapenem | < = 0.5 | < = 0.5 | < = 0.5 | NT | < = 0.5 | < = 0.5 |  |  |  |  |
| Ciprofloxacin | 0.5 | < = 0.25 | < = 0.25 | 0.5 | < = 0.25 | < = 0.25 |  |  |  |  |
| Tigecycline | < = 0.5 | < = 0.5 | < = 0.5 | < = 0.5 | < = 0.5 | < = 0.5 |  |  |  |  |
| Ampicilin-Sulbactam | > = 32 | > = 32 | > = 32 | > = 32 | < = 2 | > = 32 |  |  |  |  |
| Piperacilin-Tazobactam | > = 128 | > = 128 | < = 4 | > = 128 | < = 4 | > = 128 |  |  |  |  |
| Imipenem | 2 | NT | < = 0.25 | NT | < = 0.25 | NT |  |  |  |  |
| NT: Not tested |  |  |  |  |  |  |  |  |  |  |
| R: Resistant |  |  |  |  |  |  |  |  |  |  |
| S: Sensible |  |  |  |  |  |  |  |  |  |  |
| I: Intermedium |  |  |  |  |  |  |  |  |  |  |
|  |  |  |  |  |  |  |  |  |  |  |
|  |  |  |  |  |  |  |  |  |  |  |
|  |  |  |  |  |  |  |  |  |  |  |
|  |  |  |  |  |  |  |  |  |  |  |
