# Supplementary material for: Dissecting the inhibitory activity of Burkholderia orbicola against Gram-positive and - negative multidrug-resistant bacteria
Source: PLoS One. 2025 Jun 30;20(6):e0326906. doi: 10.1371/journal.pone.0326906 (PMC12208415; doi:10.1371/journal.pone.0326906)
Supplement: S2 Table — (DOCX) [file pone.0326906.s008.docx]

| Strain | Inhibition halo (mm) | |
| --- | --- | --- |
|  | TAtl-371^T^ | CACua-24 |
| ***Tatumella terrea* SHS-2008^T^** | 70 | 74 |
| ***Acinetobacter baumannii* strains** | | |
| 140 | 22 | 23 |
| 194 | 27 | 26 |
| 256 | 20 | 25 |
| 324 | 21 | 25 |
| 341 | 23 | 25 |
| 343 | 28 | 33 |
| 344 | 20 | 26 |
| 345 | 21 | 27 |
| 351 | 27 | 27 |
| ***Klebsiella pneumoniae* strains** | | |
| 97833 | 5 | 14 |
| 9851043 | 8 | 12 |
| 81739 | 9 | 14 |
| 945626 | 7 | 9 |
| 906667 | 10 | 10 |
| 903137 | 16 | 13 |
| ***Pseudomonas aeruginosa* strains** | | |
| 1P | 25 | 29 |
| 2P | 31 | 29 |
| 4P | 31 | 29 |
| 11P | 33 | 32 |
| 12P | 22 | 34 |
| 16P | 31 | 35 |
| 17P | 31 | 31 |
| 22P | 28 | 31 |
| 26P | 29 | 32 |
| 30P | 28 | 19 |
| ***Escherichia coli* strains** | | |
| 1 | 40 | 31 |
| 2 | 24 | 28 |
| 3 | 20 | 26 |
| 4 | 30 | 22 |
| ***Staphylococcus aureus* strains** | | |
| ATCC 25923 | 41 | 28 |
| 1 | 26 | 24 |
| 2 | 24 | 24 |
| 3 | 30 | 27 |
| 4 | 22 | 40 |

**S1 Table. Inhibitory activity of *Burkholderia orbicola* TAtl-371^T^ and CACua-24 on multidrug resistant bacteria by double-layer agar technique.**
