# Supplementary material for: Dissecting the inhibitory activity of Burkholderia orbicola against Gram-positive and - negative multidrug-resistant bacteria
Source: PLoS One. 2025 Jun 30;20(6):e0326906. doi: 10.1371/journal.pone.0326906 (PMC12208415; doi:10.1371/journal.pone.0326906)
Supplement: S3 Table — (DOCX) [file pone.0326906.s009.docx]

|  | Hexane | | Dichloromethane | | Ethyl acetate | | Methanol | |
| --- | --- | --- | --- | --- | --- | --- | --- | --- |
| Strain | Inhibition halo (mm) | | | | | | | |
|  | TAtl-371^T^ | CACua-24 | TAtl-371^T^ | CACua-24 | TAtl-371^T^ | CACua-24 | TAtl-371^T^ | CACua-24 |
| ***Tatumella terrea* SHS-2008^T^** | 13 | 44 | 10 | – | 5 | 23 | – | – |
| ***Acinetobacter baumannii*** | | |  |  |  |  |  |  |
| 256 | 6 | 9 | 9 | – | 8 | – | – | – |
| 324 | 6 | 9 | 9 | 6 | 8 | 5 | – | 6 |
| 341 | 11 | 15 | _– | 5 | – | 5 | – | – |
| 344 | 9 | 9 | 8 | – | 8 | – | – | – |
| 345 | 9 | 10 | 9 | 5 | 10 | 5 | – | – |
| ***Klebsiella pneumoniae*** | | |  |  |  |  |  |  |
| 97833 | 15 | 11 | – | 7 | – | – | – | – |
| 9851043 | 14 | 11 | – | 7 | – | – | – | – |
| 81739 | 13 | 8 | – | 8 | – | – | – | – |
| 945626 | 11 | 12 | – | – | – | – | – | – |
| 906667 | 16 | 9 | – | – | – | – | – | – |
| 903137 | 18 | 9 | – | – | – | – | – | – |
| ***Pseudomonas aeruginosa*** | | |  |  |  |  |  |  |
| 2P | – | – | – | – | – | – | – | – |
| 11P | – | – | – | – | – | – | – | – |
| 17P | – | – | – | – | – | – | – | – |
| ***Escherichia coli*** | | |  |  |  |  |  |  |
| 1 | 8 | 7 | – | – | – | – | – | – |
| 2 | 9 | 14 | – | – | – | – | – | – |
| 3 | 9 | 9 | – | – | – | – | – | – |
| 4 | 10 | 7 | – | – | – | – | – | – |
| ***Staphylococcus aureus*** | | |  |  |  |  |  |  |
| 1 | 9 | 14 | – | – | – | – | – | 8 |
| 2 | 13 | 11 | – | – | – | – | – | – |
| 3 | 15 | 10 | – | – | – | – | – | – |
| 4 | 16 | 14 | – | – | – | – | – | – |

**S2 Table. Inhibitory activity of *Burkholderia orbicola* TAtl-371^T^ extracts and CACua-24 on multidrug resistant bacteria.**
